# Rasch Analysis of the Premature Ejaculation Diagnostic Tool (PEDT) and the International Index of Erectile Function (IIEF) in an Iranian Sample of Prostate Cancer Patients

**DOI:** 10.1371/journal.pone.0157460

**Published:** 2016-06-23

**Authors:** Chung-Ying Lin, Amir H. Pakpour, Andrea Burri, Ali Montazeri

**Affiliations:** 1 Department of Rehabilitation Sciences, Faculty of Health and Social Sciences, The Hong Kong Polytechnic University, Hung Hom, Hong Kong; 2 Social Determinants of Health Research Center, Qazvin University of Medical Sciences, Qazvin, Iran; 3 Health and Rehabilitation Research Institute, Auckland University of Technology, Auckland, New Zealand; 4 Waitemata Pain Service, Department of Anaesthesia and Perioperative Medicine, North Shore Hospital, Auckland, New Zealand; 5 Mental Health Research Group, Health Metrics Research Centre, Iranian Institute for Health Sciences Research, ACECR, Tehran, Iran; University of Michigan, UNITED STATES

## Abstract

**Background:**

Male sexual dysfunction is an increasing problem across a variety of general and clinical populations, such as cancer populations; especially among prostate cancer patients who tend to receive treatments that often result in erectile dysfunction (ED) and/or premature ejaculation (PE). Therefore, in order to diagnose ED and PE in these populations, adequate and efficient instruments such as the International Index of Erectile Function 5-item version (IIEF-5) and the Premature Ejaculation Diagnostic Tool (PEDT) are needed. However, since this is an important topic additional evidence of psychometric properties of the IIEF-5 and the PEDT in such samples are required. Thus the aim of the present study was to use Rasch models to investigate the construct validity, local dependency, score order, and differential item functioning (DIF) of both questionnaires in a sample of prostate cancer patients.

**Methods:**

Prostate cancer patients (n = 1058, mean±SD age = 64.07±6.84 years) who visited urology clinics were invited to fill out the IIEF-5 and the PEDT. Construct validity was examined using infit and outfit mean square (MnSq) and local dependency using correlations between each two residual Rasch scores. Score order was investigated using step and average measures of difficulty and DIF using DIF contrast.

**Results:**

All IIEF-5 and PEDT items had acceptable infit and outfit MnSq. Step measures revealed that all but two items had disordered categories in terms of scores 1 to 3. Only one local dependency was found, and no items displayed DIF across age, educational level, and help seeking.

**Conclusions:**

The results showed that both the IIEF-5 and the PEDT had sound psychometric properties in the Rasch analyses, although some score disordering could be detected in both instruments. The results of no DIF items in both instruments suggest using them to compare ED and PE across age and educational level is adequate.

## Introduction

One in five men suffers from some sort of sexual problem, with prevalence rates showing a steady increase [1,2]. Erectile dysfunction (ED), together with premature ejaculation (PE), is the most common male sexual disorder. The largest follow-up study published on the prevalence of ED found that only 39.31% of men reported not suffering from ED, whereas 25.14% had mild ED (that is, experienced ED sometimes), 18.79% moderate ED (that is, usually experienced) and 16.77% complete ED [3]. Reported prevalences for PE range from 10% to 40% [4–6]. It is estimated that worldwide up to 322 million men will suffer from erectile dysfunction (ED) in 2025 [7]. Although ED and PE have shown to heavily impact on the quality of life of men [8], very often sufferers do not seek help from urologist or other specialists because of feelings of shame and embarrassment. In addition, many affected may not have the insight as to how serious the ED and/or PE problem is, and may resist seeking help until the sexual problem becomes extremely severe.

While prevalence rates of ED and PE are already high in general population, the estimates tend to be even higher in clinical samples, such as prostate cancer patients [9,10]. Prostate cancer and its treatments (e.g. surgery, radiation, chemotherapy) can have negative impacts on a patient’s sex life and functioning. Cancer can directly impact on sexual organs, as it is the case with prostate cancer. It also can affect body image and psycho-emotional health. In addition, side effects of cancer treatments such as fatigue, pain or anxiety can severely impact on libido and consequently affect erectile function and sexual satisfaction [9,10]. Many cancer patients may feel uncomfortable discussing the issue with healthcare professionals. In other words, they may feel and notice that their sexual function has decreased but they do not know whether this decrease should be taken serious and or may feel embarrassed to talk about the problem and actively seek for help. Therefore, there is an urgent need for validated self-report instruments that allows the patients to efficiently and privately assess their sexual function, and to decide whether seeking help is necessary. Two commonly used self-reports instruments—the International Index of Erectile Function (IIEF) for the assessment of ED [8,11] and the Premature Ejaculation Diagnostic Tool (PEDT) for the assessment of PD [12] have been designed for the above mentioned purpose but validation in prostate cancer patient population is urgently needed.

While widely applied, the evidence of the psychometric properties of both the IIEF-5 and PEDT seems insufficient because as to the best of our knowledge, the existing validation studies [8,12–14] used primarily classical test theory (CTT). CTT has the major drawback that it treats the scoring methods inappropriately (e.g., means and standard deviations), and does not differentiate the estimated parameters between items (item difficulty) and respondents (person ability) [15]. Other statistical methods for the assessment of psychometric properties such as the Rasch models are able to resolve the CTT drawbacks as they separately assesses person’s ability and item difficulty [16,17], and convert both item difficulty and person ability into a ratio scale using the identical unit called *logit*.

In addition, Rasch models investigate the issues related to the score orders, local dependency, and differential item functioning (DIF), while these issues seem to have never been investigated in both the IIEF-5 and PEDT. The score orders indicate whether the rated score reflects the respondents’ condition or not [16]. For example, a respondent who scores 1 (*very low*) on the IIEF-5 item should have less severe ED than does a respondent who scores 2 (*low*). The local dependency tests whether the IIEF-5 and PEDT items contain latent traits other than ED and PE, respectively [18]. The DIF shows that whether respondents with different characteristics (e.g., different educational level) interpret IIEF-5 and PEDT item differently [19,20].

The aim of the present study therefore was to add evidence on the psychometric properties evidence (including construct validity, local dependency, score order, and DIF) of the IIEF-5 and the PEDT in a sample of prostate cancer patients using Rasch models.

## Material and Methods

### Study population

Between March 2014 and August 2015 a sample of 1202 men who had a diagnosis of prostate cancer were invited to participate. The sample was recruited from urology clinics affiliated to medical universities in Tehran, Qazvin, Ahvaz, Guilan, and Tabriz, in Iran. Inclusion criteria were (1) aged ≥ 18 years, (2) being in a stable sexual relationship with a female partner for at least 6 months, (3) good cognitive function, and (4) voluntary participation. Sixty-three patients declined to participate, and 81 patients were not eligible because of their impaired cognitive function according to the Mini-Mental State Examination (MMSE; score ≤ 23) [21], resulting in a final sample of 1058 patients. After having their eligibility ascertained by the visiting urologist, participants were asked to complete a set of study questionnaires (see below) in a private clinic room. All patients provided written informed consent and the study protocol was approved by the Ethics Committee of Qazvin University of Medical Sciences.

### Main outcome measures

#### Five-item version of the International Index of Erectile Function (IIEF-5)

The IIEF-5 represents a short version of the 15-item version of International Index of Erectile Function used to measure erectile function [8]. The IIEF has received extensive psychometric and cross-cultural validation and translation [11,22,23], the IIEF-5 seems to be feasible because it contains only five items and has strong evidence on psychometric properties. Recently, an Iranian version of the IIEF-5 has been developed, showing good psychometric properties [14]. Each item of the IIEF-5 is rated from 1 (*very low; almost never or never; extremely difficult*) to 5 (*very high; almost always or always; not difficult*), with a lower score indicating more erectile difficulties.

In addition, the sensitivity and specificity of the IIEF-5 have been tested with satisfactory values in area under receiver operating characteristic curve (AUC). The AUC was 0.97 on a sample recruited from both the US and the UK [8]. In other words, IIEF-5 has a probability of 97% to accurately identify a man with ED or without ED.

#### Premature Ejaculation Diagnostic Tool (PEDT)

The PEDT has been designed based on the diagnostic principles of the DSM-IV-TR for PE [24]. Previous validation studies have shown satisfactory feasibility, reliability and validity of the PEDT [12]. Similarly, the recently translated Iranian version of the PEDT also showed good psychometric properties [13]. Originally, each PEDT item is rated from 0 (*not difficult at all; almost never or never; not at all*) to 4 (*extremely difficult; almost always or always*, *extremely*), with a higher score indicating more difficulties with premature ejaculation. In the present study, however, the PEDT scores were recoded from 1 (*extremely difficult; almost always or always*, *extremely*) to 5 (*not difficult at all; almost never or never; not at all*) in order to correspond to the direction of the IIEF-5 score. As a result, the higher PEDT scores in our current study indicate less ejaculation problems.

In addition, the sensitivity and specificity of the PEDT have been tested with satisfactory values in AUC. The AUC was 0.89 for PEDT on an Iranian sample [13]. In other words, PEDT has a chance of 89%.

### Statistical analysis

Descriptive analyses were conducted using SPSS 17.0 (SPSS Inc., Chicago, IL, USA). For Rasch rating scale models WINSTEPS was used [25]. Because ED and PE were considered as two separate types of sexual problems, two Rasch models were performed separately—one for IIEF-5 and another for PEDT. In addition to estimating the difficulty of each IIEF-5 item and PEDT item, we used information-weighted fit statistic (infit) mean square (MnSq) and outlier-sensitive fit statistic (outfit) MnSq to determine any redundant (infit or outfit MnSq < 0.5) or out-of-concept (infit or outfit MnSq > 1.5) item [26]. In other words, if the item fit well in its belonging construct (ED or PE), both infit and outfit MnSq should be between 0.5 and 1.5. Rasch models provide separation reliability and separation index (both included person and item separation), and a value >0.7 suggests good reliability; > 2 suggests good index [16].

The ordering of the response scores was examined using average difficulty of each response score (i.e., average measure) and step difficulty of each threshold or boundary between every two nearby scores (i.e., step measure). Satisfactory ordering of the response scores should monotonically increase average and step difficulties [27]. We also used Rasch models to test the local dependency. For that, the correlations (*r*) of the Rasch residuals between every two items were computed. That is, we examined whether some items are still correlated after the same underlying concept has been taken into account, and an *r* ≤ 0.4 is acceptable [28]. Finally, differential functioning item (DIF) for both the IIEF-5 and PEDT were tested across different age groups (Group 1: <65 years *vs*. Group 2: ≥65 years), different education level (Group 1: <6 educational years *vs*. Group 2: ≥6 years), and across help seeking (Group 1: no *vs*. Group 2: yes). According to other studies [26,29]a DIF was considered substantial when the DIF showed an absolute contrast (the difficulty for Group 1 minus the difficulty for Group 2) >0.5, meaning that the same item was interpreted in different ways by the two groups.

## Results

The mean±SD age (n = 1058), and mean diagnosis duration were 64.07±6.84, and 6.14±3.47 years, respectively. Nearly half of the participants were at stage 2 (n = 462, 43.7%) of prostate cancer and nearly half were at a medium grade at time of participation (n = 438, 41.4%) according to the Gleason grade (Table 1). The mean (SD) scores were between 2.36 (1.70) and 3.10 (1.55) in the IIEF-5; between 1.97 (1.33) and 2.66 (1.45) in the PEDT (Table 2).

**Table 1:** Demographics and clinical characteristics on participants.

**Basic characteristics**	**Mean±SD**
Age (years)	64.07±6.84
Years of education^a^	5.11±1.25
Duration-after-diagnosis (years)	6.14±3.47
Body mass index (kg/m^2^)	24.34±4.44
Depression score	7.23±4.20
Anxiety score	8.87±4.47
**Clinical characteristics**	**n (%)**
Stage at diagnosis of prostate cancer	
1	166 (15.7%)
2	462 (43.7%)
3	325 (30.7%)
Unknown	90 (8.5%)
Missing	15 (1.4%)
Gleason grade at diagnosis of prostate cancer	
Low (Score < 7)	259 (24.5%)
Medium (Score = 7)	438 (41.4%)
High (Score > 7)	248 (23.4%)
Unknown	113 (10.7%)
Diagnosis of erectile dysfunction	698 (66.0%)
Diagnosis of premature ejaculation	380 (36.1%)

^a^ With 1 missing value

**Table 2:** Observed mean score, item difficulty and fit statistics for International Index of Erectile Function-5 (IIEF-5) and Premature Ejaculation Diagnostic Tool (PEDT) (N = 1054).

Scale and item	Mean (SD)	Difficulty	Infit	Outfit
**IIEF-5** (Person/Item reliability = 0.66/0.99; Person/Item index = 1.40/10.21)				
I1: How did you rate your confidence that you could get & keep an erection?	3.10 (1.55)	−0.66	1.42	1.39
I2: When you had erections with sexual stimulation, how often were your erections hard enough for penetration?	2.93 (1.74)	−0.24	0.80	0.74
I3: During sexual intercourse, how often were you able to maintain your erection after you had penetrated your partner?	2.47 (1.66)	0.30	0.68	0.57
I4: During sexual intercourse, how difficult was it to maintain your erection to completion of intercourse?	2.36 (1.70)	0.33	1.41	1.43
I5: When you attempted sexual intercourse, how often was it satisfactory to you?	2.49 (1.67)	0.27	0.74	0.67
**PEDT** (Person/Item reliability = 0.68/0.99; Person/Item index = 1.46/9.63)				
P1: How difficult is it for you to delay ejaculation?	2.55 (1.36)	−0.18	1.18	1.17
P2: Do you ejaculate before you want to?	2.66 (1.45)	−0.45	0.99	0.95
P3: Do you ejaculate with very little stimulation?	1.97 (1.33)	0.54	1.15	1.13
P4: Do you feel frustrated because of ejaculating before you want to?	2.10 (1.44)	0.28	0.77	0.70
P5: How concerned are you that your time to ejaculation leaves your partner sexually unfulfilled?	2.45 (1.59)	−0.19	0.92	0.89

Note: The rating scale in both IIEF-5 and PEDT uses a 5-point-Likert scale with 1 represents *the worst* and 5 *the best* conditions. A higher score in each item indicates a better sexual ability.

Four participants did not respond to all the IIEF-5 and PEDT items and were therefore not included in the analyses, resulting in a sample of N = 1054 patients used for the Rasch analyses. The mean score of each item ranged from 2.36 to 3.10 for the IIEF-5 and from 1.97 to 2.66 for the PEDT (Table 2). The difficulty was −0.66 to 0.33 for the IIEF-5 and −0.45 to 0.54 for the PEDT. Infit (0.68 to 1.42 for the IIEF-5; 0.77 to 1.18 for the PEDT) and outfit MnSq (0.57 to 1.43 for the IIEF-5; 0.70 to 1.17 for the PEDT) were acceptable for all individual items. In addition to the slightly low value for person separation reliability (0.66 for the IIEF-5 and 0.68 for the PEDT), item separation reliability (0.99), person separation index (≥1.40), and item separation index (≥9.63) were all satisfactory (Table 2).

**Fig 1:**
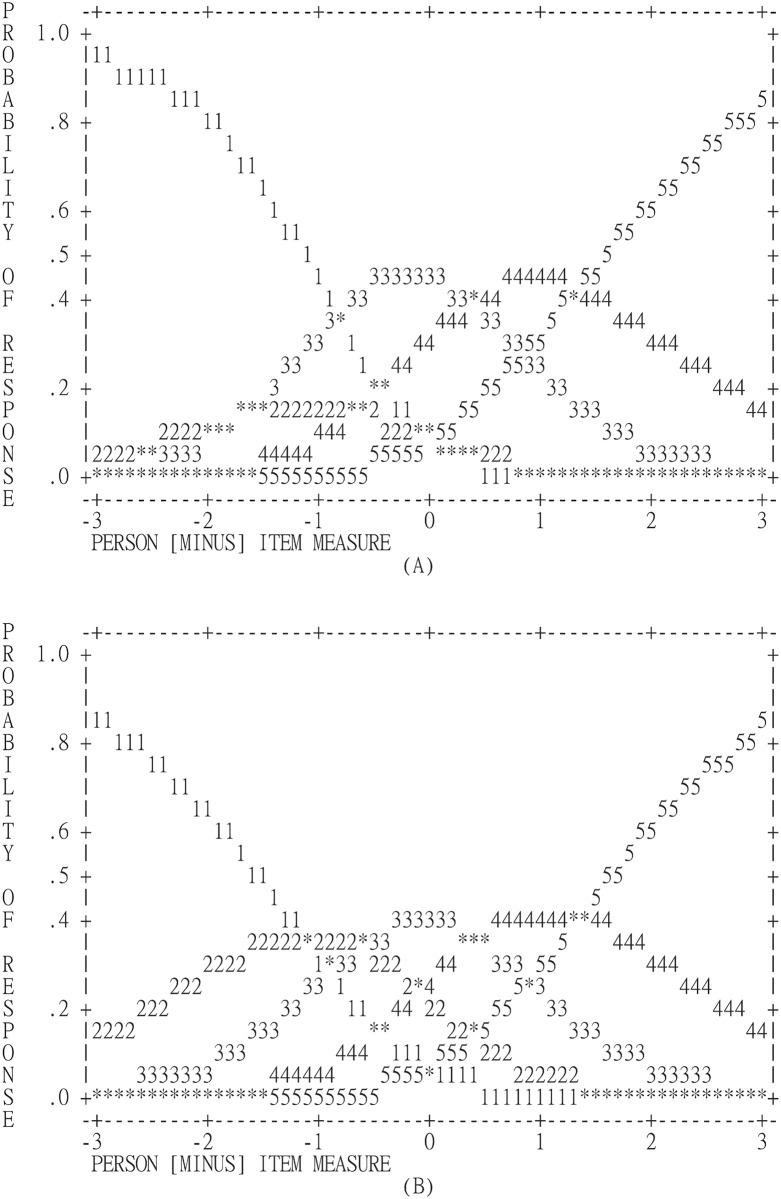
Disordering graph. (A) An example of disordering graph for IIEF-5 (Item 1); the rating scale is a 5-point-Likert scale with 1 represents *the worst* and 5 *the best* conditions. (B) An example of ordering graph for PEDT (Item 1); the rating scale is a 5-point-Likert scale with 1 represents *the worst* and 5 *the best* conditions.

Although the average measures were monotonically increased by the categories for all IIEF-5 and PEDT items, step measures revealed that all but two items (P1, P2) had disordered categories in terms of scores 1 to 3 (Table 3). The disordered pattern showed that participants intended not to select score 2 (Fig 1a), and the probabilities of rating on scores 2 to 4 were low even in the ordering items (Fig 1b).

**Table 3:** Threshold disordering tests for International Index of Erectile Function-5 (IIEF-5) and Premature Ejaculation Diagnostic Tool (PEDT).

IIEF-5 item #	Item score	Average measure	Step measure	PEDT item #	Item score	Average measure	Step measure
**I1**				**P1**			
	1 = very low	−2.77	--		1 = extremely difficult	−2.75	--
	2 = low	−1.72	−0.66		2 = very difficult	−1.27	−1.34
	3 = moderate	−0.87	−2.34		3 = moderate difficult	−0.23	−0.80
	4 = high	0.29	−0.30		4 = somewhat difficult	0.89	0.24
	5 = very high	1.98	0.66		5 = not difficult at all	2.51	1.19
**I2**				**P2**			
	1 = almost never or never	−1.61	--		1 = almost always or always	−3.04	--
	2 = a few times	−0.87	0.81		2 = more than half the time	−1.32	−1.79
	3 = sometimes	−0.38	−1.05		3 = about half the time	−0.34	−0.32
	4 = most times	0.21	−1.04		4 = less than half the time	0.50	−0.05
	5 = almost always or always	1.59	0.31		5 = almost never or never	1.80	0.34
**I3**				**P3**			
	1 = almost never or never	−1.08	--		1 = almost always or always	−1.42	--
	2 = a few times	−0.32	1.32		2 = more than half the time	−0.27	0.18
	3 = sometimes	0.17	−0.55		3 = about half the time	0.45	0.08
	4 = most times	0.76	−0.36		4 = less than half the time	1.28	0.46
	5 = almost always or always	2.10	0.79		5 = almost never or never	2.78	1.45
**I4**				**P4**			
	1 = extremely difficult	−0.93	--		1 = extremely	−1.44	--
	2 = very difficult	−0.11	0.94		2 = very	−0.39	0.23
	3 = difficult	0.34	0.28		3 = moderately	0.23	−0.03
	4 = slightly difficult	0.78	0.62		4 = slightly	0.90	0.19
	5 = not difficult	1.55	−0.52		5 = not at all	2.16	0.73
**I5**				**P5**			
	1 = almost never or never	−1.12	--		1 = extremely	−1.86	--
	2 = a few times	−0.35	1.18		2 = very	−0.79	−0.30
	3 = sometimes	0.15	−0.49		3 = moderately	−0.19	−0.17
	4 = most times	0.74	−0.32		4 = slightly	0.40	−0.27
	5 = almost always or always	2.05	0.72		5 = not at all	1.51	−0.01

Note: The rating scale in PEDT (1 represents *the worst* and 5 represents *the best* ejaculation function) is different from that in the original PEDT (0 represents *the best* and 4 represents *the worst* ejaculation function)

Only one local dependency was found for the IIEF-5, and none for the PEDT (Table 4). In addition, no substantial DIF was found in both the IIEF-5 and PEDT across age (<65 years *vs*. ≥65 years; DIF contrast = −0.18 to 0.11), educational level (<6 educational years *vs*. ≥6 educational years; DIF contrast = −0.13 to 0.13), and seeking help (no *vs*. yes; DIF contrast = −0.23 to 0.34) (Table 5).

**Table 4:** Tests of local dependency for International Index of Erectile Function-5 (IIEF-5) and Premature Ejaculation Diagnostic Tool (PEDT).

IIEF-5 Item #	IIEF-5 Item #	*r*	PEDT Item #	PEDT Item #	*r*
I1	I2	−0.09	P1	P2	−0.19
	I3	−0.25		P3	−0.30
	I4	**−0.43**		P4	−0.33
	I5	−0.23		P5	−0.29
I2	I3	−0.01	P2	P3	−0.22
	I4	−0.35		P4	−0.26
	I5	−0.20		P5	−0.34
I3	I4	−0.30	P3	P4	−0.15
	I5	−0.04		P5	−0.28
I4	I5	−0.26	P4	P5	−0.07

Absolute *r* > 0.4, which exceeds the cutoff of correlation for local dependency, is in **bold**.

**Table 5:** Tests of differential item functioning (DIF) for International Index of Erectile Function-5 (IIEF-5) and Premature Ejaculation Diagnostic Tool (PEDT).

Item # on IIEF-5	Difficulty		DIF contrast^a^	Item # on PEDT	Difficulty		DIF contrast^a^
**Test for age**	**<65 years**	**≥65 years**		**Test for age**	**<65 years**	**≥65 years**	
I1	−0.75	−0.56	−0.18	P1	−0.18	−0.18	0.00
I2	−0.19	−0.30	0.11	P2	−0.45	−0.45	0.00
I3	0.32	0.28	0.05	P3	0.49	0.61	−0.12
I4	0.33	0.33	0.00	P4	0.28	0.28	0.00
I5	0.27	0.27	0.00	P5	−0.14	−0.24	0.11
**Test for education**	**<6 years**	**≥6 years**		**Test for education**	**<6 years**	**≥6 years**	
I1	−0.60	−0.73	0.13	P1	−0.15	−0.21	0.05
I2	−0.24	−0.24	0.00	P2	−0.42	−0.50	0.07
I3	0.33	0.26	0.07	P3	0.57	0.51	0.06
I4	0.30	0.36	−0.06	P4	0.22	0.35	−0.13
I5	0.23	0.33	−0.10	P5	−0.19	−0.19	0.00
**Test for seeking help**	**No**	**Yes**		**Test for seeking help**	**No**	**Yes**	
I1	−0.66	−0.56	−0.10	P1	−0.21	0.02	−0.23
I2	−0.24	−0.20	−0.04	P2	−0.45	−0.36	−0.10
I3	0.30	0.28	0.02	P3	0.54	0.61	−0.06
I4	0.33	0.31	0.02	P4	0.32	−0.01	0.34
I5	0.27	0.22	0.06	P5	−0.19	−0.19	0.00

^a^ DIF contrasts were calculated as *logit* of age < 65 years minus *logit* of age ≥ 65 years; *logit* of educational year < 6 years minus *logit* of educational year ≥ 6 years; *logit* of not seeking help minus seeking help. For age, a positive DIF contrast indicates that those aged < 65 years had a higher item score than did those aged ≥ 65 years, and *vice versa*. The same interpretation for education and seeking help.

## Discussion

The construct validity of the IIEF-5 and PEDT has been previously confirmed by means of factor analysis using CTT methods [13,14,30], and our present results using Rasch models are in line with these results also showing satisfactory construct validity for the IIEF-5 and the PEDT, indicating the usefulness of the two instruments. However, our results additionally reveal other issues related to the questionnaires’ psychometric properties in terms of their score ordering, local dependency, and DIF items.

Except for the score ordering, all psychometric tests performed in this study suggested that both IIEF-5 and PEDT are good instruments to assess erectile and ejaculatory problems in men suffering from prostate cancer. All items fit well in their embedded ED or PE construct without noise from other unknown concepts as evidenced by our low local dependency analysis. Moreover, no items displaying DIF indicated the appropriate use of combining and comparing respondents with different demographics [31]. However, because we only tested DIF across age, education and help seeking behavior, Clinicians should use both questionnaires with caution since DIF may be different across other demographic groups. Future studies may want to further probe into this issue.

The results of the score ordering indicate that most participants preferred not choosing score 2 when filling out both the IIEF-5 and PEDT. There are several explanations to this finding. First, the patients may not have sufficient cognition to understand the descriptors of score 2. However, because we only recruited patients who had a MMSE score > 23, therefore indicating unimpaired cognition, this explanation may not be supported. Second, only few of our participants showed impaired sexual function thus, this may be reflected in the few score 2 responses. Unfortunately, we were unable to obtain the patients’ records with the clinical diagnosis to confirm this hypothesis. Third, sexual dysfunction patients may classify the problem into four levels or less. In other words, when they have a little problem on sexual dysfunction, they may consider it as no problem. Also, our data cannot answer the third hypothesis; future studies with sufficient information and solid design are warranted to examine our second and third hypotheses.

There are some limitations in the present study. First, with the available data we were unable to explore how many categories should be used in the response scores. Although our results indicated one disordering score, we are unable to make any assumption or confirm on whether a 4-point Likert scale would fit better and would show no disordering. Future studies should further investigate this issue by administering questionnaires using different response scales (e.g., 3-point *vs*. 4-point Likert scales). Second, our participants were all diagnosed with a prostate cancer, and our results may not be generalized to general population or other clinical samples. Third, we did not use any gold standard measures, including objective measures of ED [32] and PE [33], to test the validity of the IIEF-5 and PEDT. However, this was not considered a serious limitation since previous studies reported high correlations between both instruments and the Urologists’ diagnoses [5,12].

## Conclusions

Overall, the IIEF-5 and PEDT are two feasible and useful instruments for self-assessment of ED and PE. All the items fit well in their embedded construct without serious local ependency. In addition, no items displayed substantial DIF, suggesting that both instruments can be used in respondents from various demographic backgrounds. However, a certain degree of score disordering could be detected in both instruments, and future studies will need to further examine whether using a 4-point Likert scale could perform better than the current 5-point Likert scale.
